# A green wave of saltmarsh productivity predicts the timing of the annual cycle in a long-distance migratory shorebird

**DOI:** 10.1038/s41598-020-77784-7

**Published:** 2020-11-26

**Authors:** Joseph A. M. Smith, Kevin Regan, Nathan W. Cooper, Luanne Johnson, Elizabeth Olson, Ashley Green, Jeff Tash, David C. Evers, Peter P. Marra

**Affiliations:** 1 Wildlife Restoration Partnerships, Greenwich, NJ 08323 USA; 2 Biodiversity Research Institute, Portland, ME 04103 USA; 3 Smithsonian Conservation Biology Institute, Washington, DC 20007 USA; 4 BiodiversityWorks, Vineyard Haven, MA 02568 USA; 5 Eagle Hill School, Hardwick, MA 01037 USA; 6 Georgetown University, Washington, DC 20057 USA

**Keywords:** Ecology, Animal migration, Animal behaviour

## Abstract

Understanding how migratory animals respond to spatial and temporal variation in habitat phenology is critical for identifying selection pressures and tradeoffs at different life history stages. We examined the influence of breeding habitat phenology on life history timing of the eastern willet (*Tringa semipalmata semipalmata*) across a latitudinal gradient of breeding sites on the east coast of North America. To describe migration and life history timing, we deployed light-level geolocators on willets at breeding sites in New Jersey, Massachusetts and Maine, USA and evaluated additional data on life history timing and migratory connectivity from previous studies, eBird and band recoveries. Willets from Nova Scotia to Georgia winter exclusively on the Atlantic coast of northern South America and share common stopover sites. The timing of wintering site departure, breeding site arrival, nesting and southbound departure was later for birds breeding at higher latitudes while the duration of all life phases was similar across sites. Regardless of latitude, nesting corresponded with a consistent stage of seasonal salt marsh biomass accumulation and with peak spring temperature acceleration (GDD jerk). Temperature acceleration and salt marsh biomass were closely correlated with each other across the 11° latitudinal gradient we examined and with the timing of nest initiation across the northern 6° of this gradient. For this northern 6° of latitude, these results suggest that the timing of migration and breeding events in the annual cycle of eastern willets is constrained by a phenological “green wave” of spring salt marsh productivity at breeding sites.

## Introduction

The timing of life history events in species occupying seasonal environments is governed by the interaction of internal time-keeping mechanisms and external cues^1^. External cues such as day length and temperature provide a reliable indicator for species in their attempts to optimally-time life history events to maximize survival and/or reproduction^2^. While much attention is focused on phenological trends over time as the climate changes^3^, variation in life history timing can also be driven by geographic variation in environmental cues^4^. Identifying both temporal and geographic variation in these cues and phenological outcomes is critical for understanding the response of species to climate change^5,6^.

As migratory species progress through stages of their annual cycle, in many cases migrating between tropical and temperate latitudes, movements are timed precisely to coincide with seasonal resources at widely scattered and disparate locations^7–9^. The timing of life history events result from the interplay of physiological constraints^10^ and environmental factors^11^ that facilitate and limit the timing and duration of these events. Within populations, the timing of seasonal movements may vary due to a range of possible factors including intraspecific competition^12^, genetic variation^13^, access to resources^14,15^, differences in an animal’s ultimate destination^16,17^ and environmental conditions^18^. Across this variation, there is likely an optimal timing for each life stage that maximizes survival and/or reproduction. For example, in migratory birds, reproductive success is often maximized when arrival on breeding grounds is as early as conditions allow so that individuals can acquire the high-quality breeding territories ahead of competitors^19^.

Species with breeding ranges that span latitude- or elevation-driven gradients in climate may encounter a corresponding gradient in the seasonal timing of resource availability. This in turn can affect the optimal timing of life history events^16,17,20–22^. Examining the behavioral flexibility of species in adjusting the timing of life history events across the annual cycle in accordance with climatic gradients may ultimately reveal how species can or cannot respond to changes in climate across time. In order to achieve such insights, we must identify the key environmental factors that influence the timing of events as well as their location in space and time across the annual cycle.

Latitudinal gradients are known to affect the phenology of plants, particularly during spring green-up. Likewise, nesting phenology of birds may also correlate with latitude^20,23^, but the effect of such phenological gradients on the timing of preceding and subsequent stages of the annual cycle for these species is not well understood^16,24^. Staggered life history timing associated with phenological gradients across a landscape may carry over into subsequent periods of the annual cycle creating a “domino effect”^25^ where the timing of one stage dictates the timing of a subsequent stage. Alternatively, the timing of subsequent annual cycle stages can be decoupled from the timing of a prior stage. This can occur when timing is dictated by synchrony in phenology and resource availability that drives members of the population to converge on precisely-timed pulsed resources at a single place and time^26,27^. Therefore, variation in timing due to gradients in habitat phenology may result in life-history tradeoffs if optimal timing with local habitat phenology at one stage conflicts with optimal timing at a subsequent life stage^28^.

In this study, we examine how the timing of life history events varies across a latitudinal gradient in a long-distance migratory shorebird, the eastern willet (*Tringa semipalmata semipalmata*), breeding in salt marsh habitats from Georgia to Maine, USA. Salt marshes are an ideal model for exploring the influence of habitat phenology on constituent species because marshes are composed of a limited suite of plant species^29^ and occupy a vast temperature gradient across latitude from maritime Canada south to the Gulf of Mexico^30^. Salt marshes respond to this gradient by having higher productivity and longer growing seasons in southern regions compared to a compressed growing season in northern parts of its range^31^. Eastern willets occupy nearly the entire range of salt marsh in eastern North America^32^. This allows for the examination of how latitude-driven variation in habitat phenology impacts the timing of events throughout the annual cycle.

Because little is known about the migration and nonbreeding range of eastern willets, we first describe the migration system and wintering destinations of breeding willets on the east coast of North America using light-level geolocators (hereafter geolocators) and band recovery data. We then examine data on the timing and duration of life cycle events using data from geolocators, eBird and previous studies^33,34^ to describe latitudinal variation in the timing of the willet nesting season and to determine whether this variation has carry over effects on the timing of subsequent life history events. Finally, we derive two independent indicators of salt marsh phenology based on temperature and productivity to document latitudinal patterns of salt marsh phenology. Using these phenological indicators, we test the hypothesis that the timing of events throughout the eastern willet annual cycle is tightly linked to a phenological “green wave” at breeding sites where nest initiation occurs at the same seasonal stage of *Spartina* productivity across a latitudinal gradient.

## Methods

### Geolocator deployment

**Figure 1:**
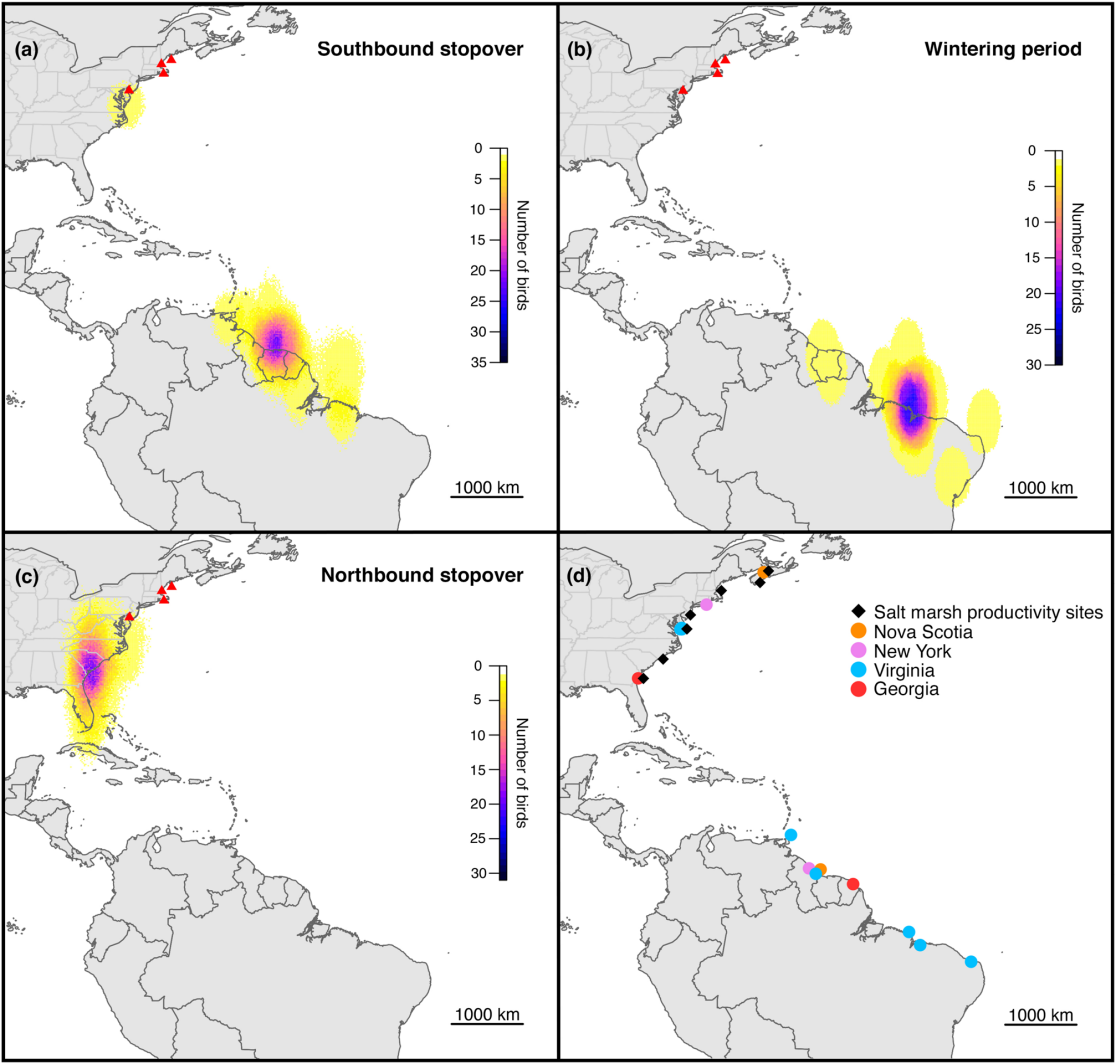
Map depicting geolocator deployment sites (red triangles) and the estimated locations of (**a**) southbound stopover, (**b**) wintering, and (**c**) northbound stopover sites from light-level geolocator data. Panel d shows locations of previous eastern willet breeding studies^34,76^, sites of previous *Spartina alterniflora* productivity studies, and band recovery data outside of the breeding range. Each panel (**a**–**c**) was created by combining the 95th quantiles of estimated positions from each time period and colors indicate the number of birds overlapping in space. Sample sizes vary because individuals tracked twice wintered in nearly identical locations but differed in their southbound and northbound stopover locations, and because some tags (n = 6) stopped working in late winter.

The eastern willet breeds along the Gulf and Atlantic coasts of eastern North America and nests primarily in salt marsh and adjacent dune habitats and exhibits high breeding site fidelity^34^. From 2010 to 2014, we captured willets and attached geolocators that recorded light levels and wet-dry data to leg bands (BAS MK18L or Intigeo-C65) at salt marsh study sites (Fig. 1a,d) during the breeding season between April and July.

in New Jersey (Gandy’s Beach: 39.27°, − 75.24°, n = 30 deployed in 2010, 30 deployed in 2011), Massachusetts (Parker River NWR: 42.77°, − 70.80°, n = 3 deployed in 2011; n = 3 deployed in 2012; n = 4 deployed in 2014), Martha’s Vineyard: 41.33°, − 70.01°, n = 4 deployed in 2013, n = 4 deployed in 2014)) and Maine (Rachel Carson NWR: 43.32°, − 70.56°, n = 5 deployed in 2011; n = 3 deployed in 2012; n = 4 deployed in 2014). Due to climate similarities and close geographic proximity (60 km, mean peak spring temperature acceleration date, described below, within 2 days of each other), all analyses consider geolocator data from the Parker River and Rachel Carson sites as one site (hereafter referred to as “Massachusetts north and Maine ”). We hereafter refer to the Martha’s Vineyard, MA site as “Massachusetts south”.

Incubating males and females (sex determined via size and behavior^32^) were captured on the nest with either a mist net dropped on top of the incubating bird or with a remotely operated bow net or whoosh net set at the nest^35^. Geolocators were then placed on willets using a leg-mounted color band^36^. In subsequent breeding seasons we recaptured returning birds with geolocators using similar techniques or we used a whoosh net^37^ with decoy and playback.

### Migration and life history timing from geolocators

We analyzed raw light data from the geolocators with the Solar/Satellite Geolocation for Animal Tracking package (SGAT)^38^, for Program R^39^ following guidelines laid out by Lisovski et al.^40^. SGAT uses Markov Chain Monte Carlo (MCMC) simulations to estimate twice daily locations and their associated error. We first imported raw light data via the package “GeoLight”^41^, and then used the preprocessLight and twilightEdit functions in package “TwGeos”^42^, to convert raw light data to twilight times and remove large outliers (> 35 min difference in twilight times compared to 2 surrounding days). Using a threshold of 1, we determined the appropriate zenith angle and position error for a subset of the light data. For this calibration step, we used 2–4 weeks of data during the breeding season when individuals were in a known location and not actively incubating^43^. Using a single zenith angle for the entire year, we created an initial set of locations using the thresholdPath function in SGAT to serve as an informed prior in the later MCMC analysis. If plotting of this initial path indicated biologically impossible stopover and winter locations, such as wintering in the ocean rather than on the coast, we used the Hill-Ekstrom calibration to estimate the zenith angle for southbound stopover and winter locations^40^. For each individual, we specified a model that included: (1) the initial locations derived above, (2) a distribution of errors between known and estimated locations at the site of geolocator deployment, (3) a distribution of plausible flight speeds, and (4) a list of zenith angles to be used for each day. Using three independent chains, we ran the model 60,000 times for burn-in and tuning, and a final 15,000 times to define the posterior distribution.

We determined the timing of migration and stationary nonbreeding periods using both light-level derived location data and conductivity data, which recorded when the leg-mounted light sensor was immersed in salt water^44^. Using methods described by Battley and Conklin^45^, we identified periods when willets were migrating that showed extended dry periods uncharacteristic of typical wet-dry signatures observed during sedentary periods^46^. We used this timing information along with position estimates from the posterior distribution of the MCMC simulations to define the locations of the stopover and wintering periods as the 95^th^ quantiles of positions during each period^47^. Due to little variation in day length, accurate estimation of latitude near the spring and fall equinoxes is not possible, and this effect is magnified close to the equator where there is already little variation in day length^48^. Stopover periods on southbound migration typically ended about 30 d before the fall equinox and coastal habitat at stopover locations is primarily arranged on an east/west axis. Thus, the fall equinox did not affect our estimates of southbound stopover locations. To maximize the accuracy of our nonbreeding location estimates, we excluded positions estimated 40 d before and after each equinox. Northbound migration typically began shortly after the spring equinox and therefore our latitudinal estimates of northbound stopover locations have more error (see Fig. 1c) and should be interpreted with some caution. However, the timing of migration and the stopover and wintering periods should not be affected by the equinoxes because longitude can still be estimated accurately during the equinoxes and timing was corroborated by the conductivity data. Finally, because the leg-mounted sensor is obscured during incubation of eggs^49^, we considered dark periods during daylight hours to indicate periods of incubation^50^.

We recovered geolocators from 30 individuals between 2010 and 2016 (New Jersey n = 19, Maine n = 3), Massachusetts north n = 3, Massachusetts south n = 5). Geolocators collected up to 2 years of data. For estimation of stopover and wintering locations we included both years of data for individuals captured twice if they used different areas each year. Therefore, sample sizes differ for southbound stopover, wintering, and northbound stopover locations. For analyses of schedule timing and duration we included the additional years of data from individuals that were tracked twice (New Jersey n = 4, Massachusetts south n = 5, Massachusetts north and Maine n = 2). We accounted for repeated measures of individuals by using linear mixed-effects models^51^ with individual as a random effect and deployment site as a fixed effect to test for differences among sites in timing and duration of life cycle events^52^. Estimates of timing (marginal means) by site were then used for regressions with latitude as a predictor of life-history events.

### Migration and life history timing from other sources

For additional migration information beyond our study sites, we queried band recovery records held by the United States Geological Survey Bird Banding Laboratory and Canadian Wildlife Service for eastern willets banded in the United States and Canada. We constrained our final data set to recoveries that occurred outside of the breeding range.

We compiled additional data on willet nesting phenology at other latitudes from published studies of willets in Georgia (31.99°, − 80.89°)^33^ and Virginia (37.80°, − 75.52°)^34^. For the Georgia study site, the data comprises 91 nests over a 50-year time span (1907–1955), while the Virginia study is based on 226 nests over a 3-year timespan (1977–1979). We estimated mean arrival and nest initiation dates for these studies by using the normal distribution of arrival and nest initiation data from our New Jersey study site (n = 20) and aligning the left tail of the distribution to the earliest date reported by these studies. Arrival in New Jersey occurred across a 12-day span and nest initiation occurred across a 9-day span. This approach risks biasing the estimate early if the earliest observations are outliers, but our geolocator-derived data did not reveal any outliers of this kind and breeding synchrony was also noted in the Virginia study^34^. We combined these published data with our own timing data (derived from geolocators) to calculate a linear regression to examine the effect of latitude on the timing of breeding site arrival and nest initiation.

### Arrival timing from eBird observations

**Figure 2:**
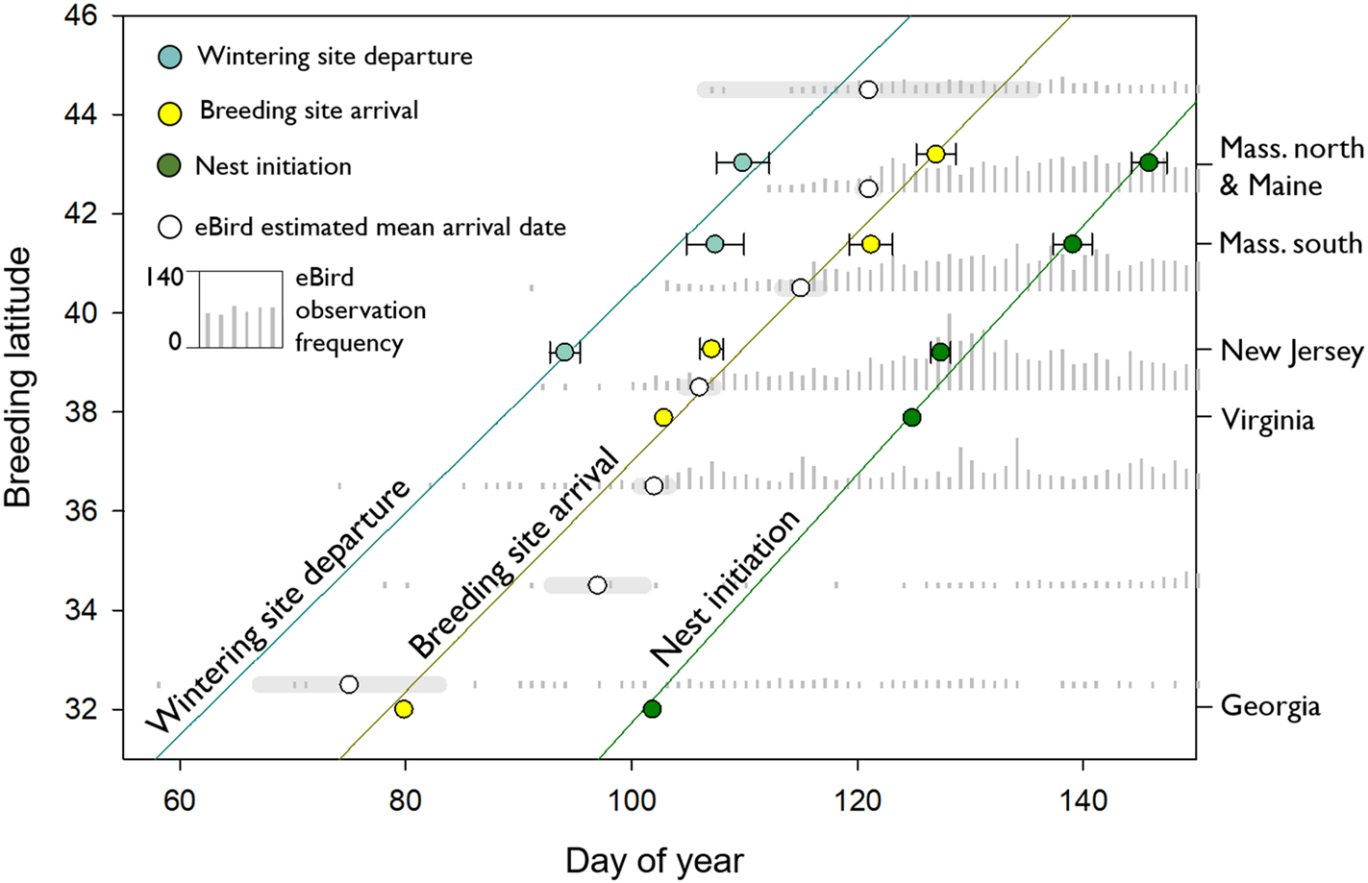
Latitudinal gradient in the timing of eastern willet northbound wintering site departure, breeding site arrival and nest initiation timing with SE bars around geolocator-derived estimates. Regression lines are based on timing data derived from geolocators and previous studies. eBird observations of eastern willets are displayed as histograms by 2-degree latitude bins. Estimated mean arrival date derived from eBird is displayed for each bin along with SE bars depicting variation among years.

As an independent measure of arrival timing during northbound migration across the latitudinal gradient, we examined patterns of willet observations in eBird^53^ data downloaded September 2018. We constrained eBird data to east coast states and Canadian provinces only, grouped into two-degree latitude bins (n = 7 bins), and only used observations that explicitly identified the eastern willet subspecies. We did not include count data, only the frequency of discrete observations per day, per latitude bin (Fig. 2). To estimate arrival timing from eBird data, we defined mean arrival as that date when a cumulative 2% of the seasonal total of discrete observations were reported on eBird over a 10-year span (2008–2017). This 2% metric matched the mean arrival date from our NJ geolocator dataset. We used the NJ data as a reference because it had the highest geolocator sample size as well as the highest number of eBird observations (38.5° bin, n = 4224). Observations were sparse in the two southern bins (32.5°, n = 259; 34.5°, n = 286) as well as in the northern-most bin (44.5°, n = 134), but were more frequent in the central portion of the study area (36.5°, n = 1934; 40.5°, n = 4031; 42.5°, n = 2499).

### Temperature gradients and salt marsh phenology

To test the role of temperature as the ultimate determinant of both *Spartina* and willet phenology, we examined 30-year average cumulative Growing Degree Days (GDD) across a 14° latitudinal gradient along the east coast of North America. Cumulative GDD are the sum of mean daily temperatures above a temperature threshold and have widespread use in studying and predicting phenology^54^. We used the website pnwpest.org^55^ to calculate 30-year average (1981–2010) cumulative GDD with data from the National Climate Data Center using the single sine method^56^ with a minimum temperature threshold for *Spartina* growth of 10 °C^31^. We did this for the nearest weather station to each of our study sites, those of previous willet nesting studies in Virginia and Georgia and a series of 26 additional coastal locations along the latitudinal gradient (32°–44°) to establish the relationship between GDD and latitude. To determine relative change in GDD over time, we also calculated the 10 year average (2006–2015) for our study sites and that of previous willet nesting studies and used a matched-pairs t-test to determine whether the date of peak spring temperature acceleration has shifted over time.

Once cumulative degree days were calculated we took the third derivative of this value, Growing Degree Day Jerk, which represents temperature acceleration^57^. In temperate environments temperature acceleration reaches a peak in early spring and this peak is frequently considered a proxy for “onset of spring”^58,59^. We examined the correlation between these peaks, our model of salt marsh growth phenology (described below) and nest initiation dates for sites across the latitudinal gradient. For these analyses we used the 10-year average for contemporary geolocator-derived data and the 30-year average for the older Georgia and Virginia data^34^.

### Salt marsh productivity phenology

To examine whether the timing of breeding site arrival and nest initiation is correlated with the plant phenology of breeding habitats, we compiled results from eight studies of salt marsh productivity (*Spartina alterniflora* biomass) to model the effect of latitude and date on the seasonal growth dynamics of *S. alterniflora*, the dominant plant species in salt marshes along the east coast of North America. Willets primarily nest in salt marsh (composed of *S. alterniflora*^60^, *Spartina patens* and *Distichlis spicata*), transitional areas between marsh and upland and adjacent upland habitat such as dunes^34^. We chose to use *S. alterniflora* as a breeding habitat proxy because of the prevalence of studies on the growth of this species^61^ and the fact that the seasonal growth of salt marsh plant species are closely correlated^62,63^.

To model seasonal growth, we selected studies that measured *Spartina* productivity on at least a monthly basis from late winter through summer. We further constrained our inclusion criteria to only those studies that gave exact dates for measurements. This resulted in a set of eight studies conducted across a broad latitudinal range (Fig. 1a, 31.33°–45.3°) in Georgia^64,65^, North Carolina^66^, Virginia^67^, New Jersey^68^, Massachusetts^69^ and Nova Scotia^70,71^ between the years 1966–1992. Depending on the study, we extracted mean and variance data either from tables or from figures using Web Plot Digitizer^72^.

Because *Spartina* productivity varies widely across years^73^ and latitudes^31^, we converted biomass measurements to percent of total seasonal biomass to standardize the data. We then conducted a meta-analysis that accounted for within and between study variance using an inverse-variance weighted fixed-effect in a generalized estimating equation which also accounted for repeated measures within studies^74^. The model was fit via linear regression and identity link function using the AR(1) working correlation structure and diagnostics confirmed that residuals met assumptions of normality. We examined the effect of latitude, date, and a latitude * date interaction to predict Spartina growth stage during early stages of green up. We used the resulting model to determine the mean stage of biomass accumulation at each site across all nesting initiation dates derived from our geolocator dataset. To test for a threshold effect in the relationship between phenological indicators and the predicted date of nest initiation, we used cutpoint analysis via the maxstat_test function in the Coin package^75^ in program R^39^. The maxstat_test defines a single cutpoint in the independent variable and generates a Tmax statistic for this partition which represents the maximum observed T statistic for all possible two-group partitions of the data.

## Results

### Migratory patterns and connectivity

We recorded 41 southbound migrations from 30 individuals. When departing the breeding grounds, 40 of 41 individuals (98%) made a direct transoceanic flight to northern South America, while one bird (2%) first flew southwest from Maine to a stopover site near the Chesapeake Bay, Maryland, before flying to South America. Of the 40 individuals flying directly to South America, 9 (22%) proceeded directly from the breeding grounds to their stationary nonbreeding (hereafter wintering) site, while 31 (78%) made an initial stopover on the northern coast of South America, primarily in Guyana, Suriname, or French Guiana (Fig. 1a). Of these 31 birds, 28 (90%) flew directly to their wintering site after their stopover, while 3 (10%) made an additional stopover before arriving to their wintering site. Willets remained on stopover for approximately 2 weeks (Table 1).

**Table 1:** Timing of eastern willet annual cycle periods (marginal means from linear mixed model that accounts for repeated tracking of some individuals).

	NJ	MA south	MA north and ME	NJ versus MA south	NJ versus MA north and ME	MA south versus MA north and ME
	Mean day of year ± SE	Mean day of year ± SE	Mean day of year ± SE	*p* value	*p* value	*p* value
Latitude	39.2	41.38	43.03			
Fall migration departure	(Jul 11) 192.3 ± 2.8, n = 23	(Aug 3) 215.2 ± 5.1, n = 10	(Jul 26) 207.1 ± 4.9, n = 8	0.002	0.039	0.5
Winter arrival	(Aug 4) 215.7 ± 3.8, n = 23	(Aug 25) 236.8 ± 6.7, n = 6 (231.4 ± 5.9, n = 10)	(Aug 20) 232.2 ± 7.5, n = 5 (223.7 ± 6.2, n = 8)	0.019	0.015	0.5
Spring migration departure	(Apr 4) 94.1 ± 1.3, n = 21	(Apr 17) 107.4 ± 2.6, n = 6	(Apr 20) 109.9 ± 2.3, n = 7	< 0.0001	0.0003	0.76
Breeding site arrival	(Apr 17) 107.1 ± 1.0, n = 21	(May 1) 121.2 ± 1.9, n = 6	(May 7) 127.0 ± 1.7, n = 7	< 0.0001	< 0.0001	0.097
Nest initiation	(May 7) 127.4 ± 0.9, n = 20	(May 19) 139.1 ± 1.7, n = 6	(May 26 ) 145.8 ± 1.6, n = 7	< 0.0001	< 0.0001	0.019

Statistical comparison of the timing of different periods of the annual cycle for eastern willets tracked with geolocators from breeding sites in New Jersey (NJ), Massachusetts south (MA south) and Massachusetts north and Maine (MA north and ME). For winter arrival, summary info is presented for the subset of individuals that made a stopover during southbound migration and, in parentheses, all individuals including those that migrated directly to wintering sites. Tests for the winter arrival phases compare the subset that made a southbound stopover.

All tracked eastern willets spent the winter along the coast of northern South America (Fig. 1b). Individuals that had 2 years of tracking data (n = 11) wintered in the same general location each year and therefore we only display results from the first winter for each individual (n = 30). The majority (87%, 26 of 30) wintered along a ~ 1200 km stretch of coastline in Brazil with many winter location estimates centered near the Baía de São Marcos in Maranhão state, while two individuals (7%) wintered on the eastern tip of Brazil, and two individuals (7%) wintered along the coast of Guyana, Suriname, or French Guiana.

Sample sizes for northbound migration were smaller because some tags (n = 6) stopped working during the winter. We were not able to estimate the location of northbound stopover sites for three additional individuals because latitudinal variation in our location estimates was too large. In March and April, all willets departing northbound from wintering sites in South America again made a direct transoceanic flight, making landfall along the southeast coast of the U.S. in Florida, Georgia or South Carolina (Fig. 1c). After a roughly 2-week stopover there (Table 1), they proceeded north to their previous year’s breeding sites in New Jersey, Massachusetts, and Maine.

All prior band recoveries of eastern willet (n = 8 between 1957 and 1998) were made on the northern coast of South America in Venezuela, Guyana, Suriname, French Guiana, and Brazil during the stopover and wintering period between June (when southbound migration from breeding sites begins) and March. Five of these recoveries were of dead birds (at least 3 of these were shot), one was captured and released, one with color bands was resighted and the disposition of the bird in remaining encounter is unknown. These birds originated from breeding sites in Nova Scotia, New York, Virginia, and Georgia (Fig. 1a). Six of these recoveries were from birds that were part of the two studies we drew upon for additional willet arrival and nest timing data (VA, n = 5 and GA, n = 1). Thus, evidence from both light-level geolocators and band recoveries indicates that birds breeding at sites across the east coast of the U.S. have highly overlapping wintering locations in South America.

### Duration and timing of annual cycle stages from geolocators

Overall, the duration of each stage of the annual cycle was similar in length regardless of breeding location (Table 2). There is a potential trend toward shorter stopovers during northward migration and longer stopovers during southbound migration for more southern-breeding individuals. The only significant comparison supporting this pattern was a longer northbound stopover period for Massachusetts north and Maine birds compared with New Jersey birds (Table 2). Migration between North and South America for both northbound and southbound flights consisted of a single sustained transoceanic flight encompassing approximately 4 days (southbound migration average duration, 4.26 ± 2.78 days; northbound, 4.21 ± 1.89 SE days).

**Table 2:** Duration of eastern willet annual cycle periods (marginal means from linear mixed model that accounts for repeated tracking of some individuals).

	NJ days ± SE	MA south days ± SE	MA north and ME days ± SE	NJ versus MA south	NJ versus MA north and ME	MA south versus MA north and ME
				*p* value	*p* value	*p* value
Latitude	39.2	41.38	43.03			
Fall migration and stopover duration	19.7 ± 2.2, n = 23	16.2 ± 3.5, n = 10	16.1 ± 3.7, n = 8	0.68	0.69	0.99
Wintering period	241.6 ± 3.5, n = 21	236 ± 6.6, n = 6	249 ± 6.1, n = 7	0.76	0.59	0.38
Spring migration and stopover duration	12.4 ± 0.68, n = 21	14.0 ± 1.3, n = 6	17.3 ± 1.2, n = 7	0.54	0.004	0.17
Breeding site pre-nesting period	20.4 ± 1.1, n = 21	17.8 ± 2.2, n = 6	18.9 ± 2.0, n = 7	0.51	0.77	0.92
Nesting period	64.5 ± 2.9, n = 21	78.1 ± 5.7, n = 6	61.8 ± 5.2, n = 7	0.10	0.89	0.11

Statistical comparisons of the duration of different periods of the annual cycle for eastern willets tracked with geolocators from breeding sites in New Jersey (NJ), Massachusetts south (MA south) and Massachusetts north and Maine (MA north and ME). Sample size differences are a result of geolocators that failed prematurely.

The timing of life history events in willets breeding at the most southern site, New Jersey, was significantly earlier for all stages by an average of 15 days when compared with the birds originating from Massachusetts and Maine (Fig. 2, Table 1). This includes southbound migration departure from breeding sites, northbound migration departure from wintering sites, breeding site arrival and incubation initiation. Comparing south Massachusetts with Massachusetts north and Maine sites, the timing of life history events trended earlier for Massachusetts south (with nest initiation significantly earlier) for all stages except southbound departure (Table 1). Given that duration is consistent among sites, this indicates that events in the annual cycle are consistently shifted later for individuals breeding at higher latitudes.

### Additional evidence for a latitudinal gradient in annual cycle timing from breeding site studies and eBird

Across 5 study sites spanning an 11° latitudinal gradient, we found increasingly later breeding site arrival timing (Fig. 2, R^2^ = 0.99, df = 1, *p* = 0.004, Table S1) and nest initiation timing (Fig. 2, R^2^ = 0.99, df = 1, *p* = 0.004, Table S1) with increasing latitude. Although they have different intercepts (i.e. event timing), the slopes of the arrival and breeding date regressions described above were not significantly different from each other (arrival/breeding category * date interaction, *p* = 0.33, Fig. 2, Table S1), reinforcing the pattern of stable duration of life cycle events across populations from different breeding latitudes despite variable latitude-dependent timing (Table 2). An independent estimate of breeding site arrival timing across latitude from the eBird 10-year aggregate dataset suggested a similar latitudinal gradient in migration arrivals (Fig. 2, R^2^ = 0.89, df = 1, *p* = 0.001, n = 7, Table S2).

### Indicators of latitudinal variation in breeding habitat phenology: temperature

When comparing the 10-year average peak temperature acceleration date to the 30-year average, the “onset of spring” had shifted significantly earlier by an average of 3.6 days across all sites (matched pairs t = 4.96, DF = 6, *p* = 0.003). For the contemporary data from New Jersey, Massachusetts and Maine sites, mean nest initiation date was within ± 4.25 days of the 30-year average date of peak spring temperature acceleration and within ± 1.6 days of the 10-year average. For the previous study in Virginia^34^, mean nest initiation date was within 2 days of the 30-year average temperature acceleration peak (and 4 days for the 10-year average). For the prior study in Georgia^76^ the mean nest initiation was not closely associated with the average peak temperature acceleration, occurring 13 days later for the 30-year average and 18 days later for the 10-year average.

**Figure 3:**
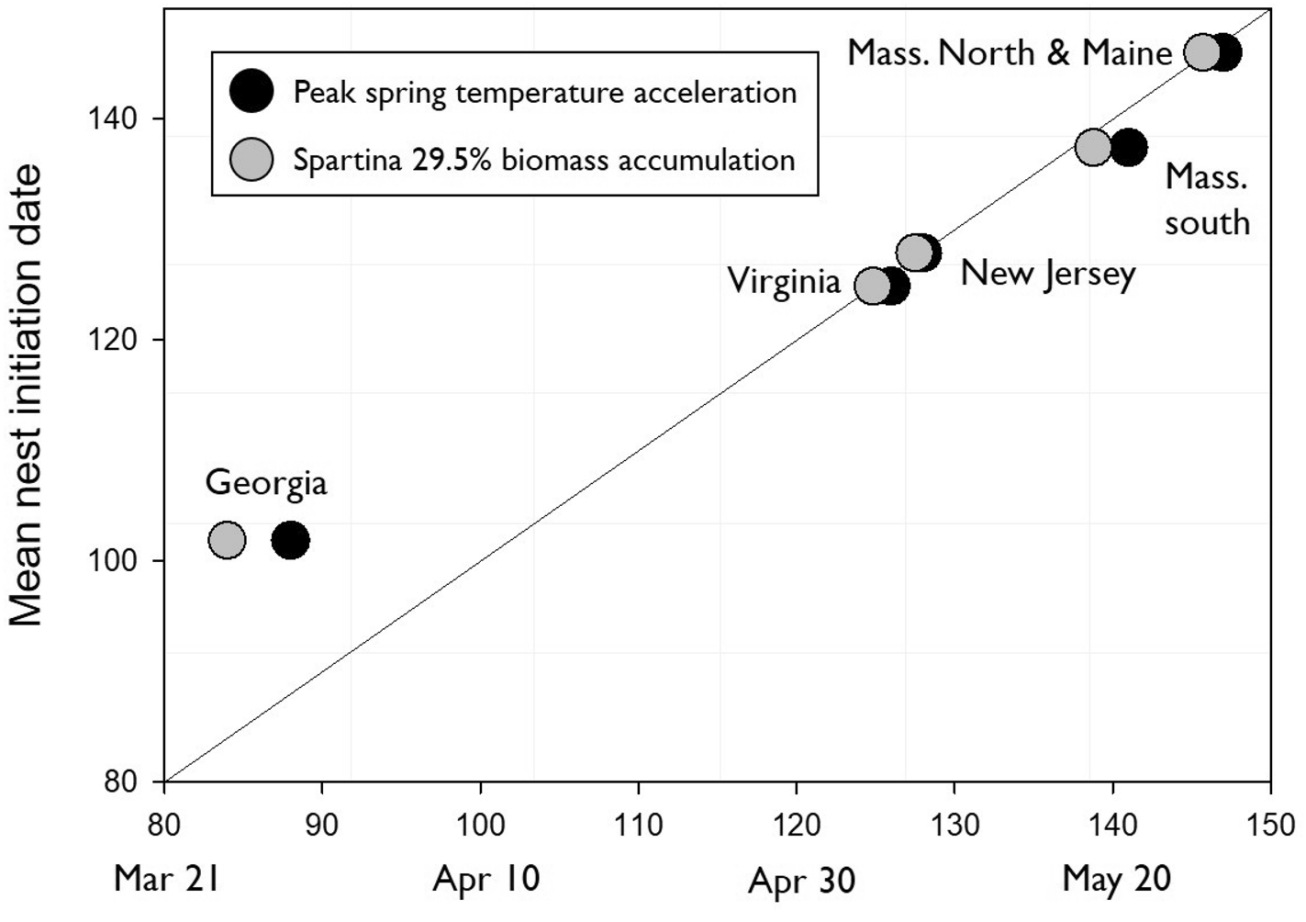
The relationship between willet nest initiation date at four sites and two phenological indicators: the date of peak spring temperature acceleration (GDD jerk) and the date when Spartina biomass accumulation has reached 29.5% (corresponding to the mean stage of biomass accumulation across all nest initiation dates derived from geolocators). The diagonal line indicates a 1:1 relationship between phenological indicator date and nesting date.

Nest timing across all sites was correlated (slope = 0.73, R^2^ = 0.98, df = 1, *p* < 0.0013, Table S3) with the date of peak spring temperature acceleration (the “onset of spring”, GDD Jerk). If Georgia is removed from the analysis, the peak spring temperature acceleration date predicted mean nest initiation timing nearly to the day (slope = 0.99, R^2^ = 0.98, df = 1, *p* < 0.0011, Fig. 3, Table S4).

### Indicators of latitudinal variation in breeding habitat phenology: spartina biomass production

The timing of salt marsh production toward maximum seasonal biomass varied by date and latitude. A Generalized Estimating Equation linear model that included date, latitude, and an interaction between these two variables indicated that biomass accumulation begins earlier in the season at more southern latitudes and the slope increases with increasing latitude (Table 3).

**Table 3:** Model results for *Spartina* spring green-up.

Independent variable	Estimate	SE	Chi-square	*p* value
Intercept	3.103	0.39	63.66	< 0.0001
Date	− 0.015	0.0038	15	< 0.0001
Latitude	− 0.096	0.0079	143.93	< 0.0001
Date * latitide	− 0.001	0.000085	41.55	< 0.0001

Results from a repeated-measures generalized estimating equation of the influence of date and latitude on the seasonal progress of *Spartina alterniflora* productivity. Data are derived from eight studies of *Spartina* productivity along a latitudinal gradient from Georgia to Nova Scotia (see text for references).

Willet mean first nest initiation date, defined by the onset of egg incubation based on geolocator data, corresponded to a specific modeled growth stage of *Spartina*, with nest initiation occurring at a mean 29.5% of total seasonal *Spartina* biomass regardless of latitude. This was due to a time-shifted nesting phenology across the latitudinal gradient. There was no significant difference in *Spartina* phenological stage at nest initiation among the three sites (Wilcoxon X^2^ = 3.8, DF = 2, *p* = 0.15, Fig. 3; New Jersey mean = 27.5%, n = 21; Massachusetts south mean = 29.2%, n = 6; Massachusetts north and Maine mean = 30.3%, n = 7), indicating that nesting occurred at the same phenological stage regardless of latitude.

For previous willet nesting studies, this pattern remained consistent for Virginia, with salt marsh growth phenology at the mean nest initiation date similar to the New Jersey, Massachusetts and Maine study sites (29.5% of total seasonal biomass, t = -1.69, DF = 2, *p* = 0.23, 95% CI 26.1–33.9%). In Georgia, the predicted stage of salt marsh progress was significantly greater than the other sites (37.9% of total seasonal biomass, t = -13.27, DF = 2, *p* = 0.006, 95% CI 34.1–45.8%). A regression including all sites that used the 29.5% biomass accumulation date to predict mean nest initiation date showed a strong correlation (slope = 0.69, R^2^ = 0.97, df = 1, *p* < 0.002, Table S5) and predicted mean nest initiation timing nearly to the day (slope = 1) when Georgia was removed from the analysis (slope = 1.03, R^2^ = 0.99, df = 1, *p* < 0.0003, Fig. 3, Table S6).

### Nest timing, spring temperature and modeled salt marsh phenological stage

**Figure 4:**
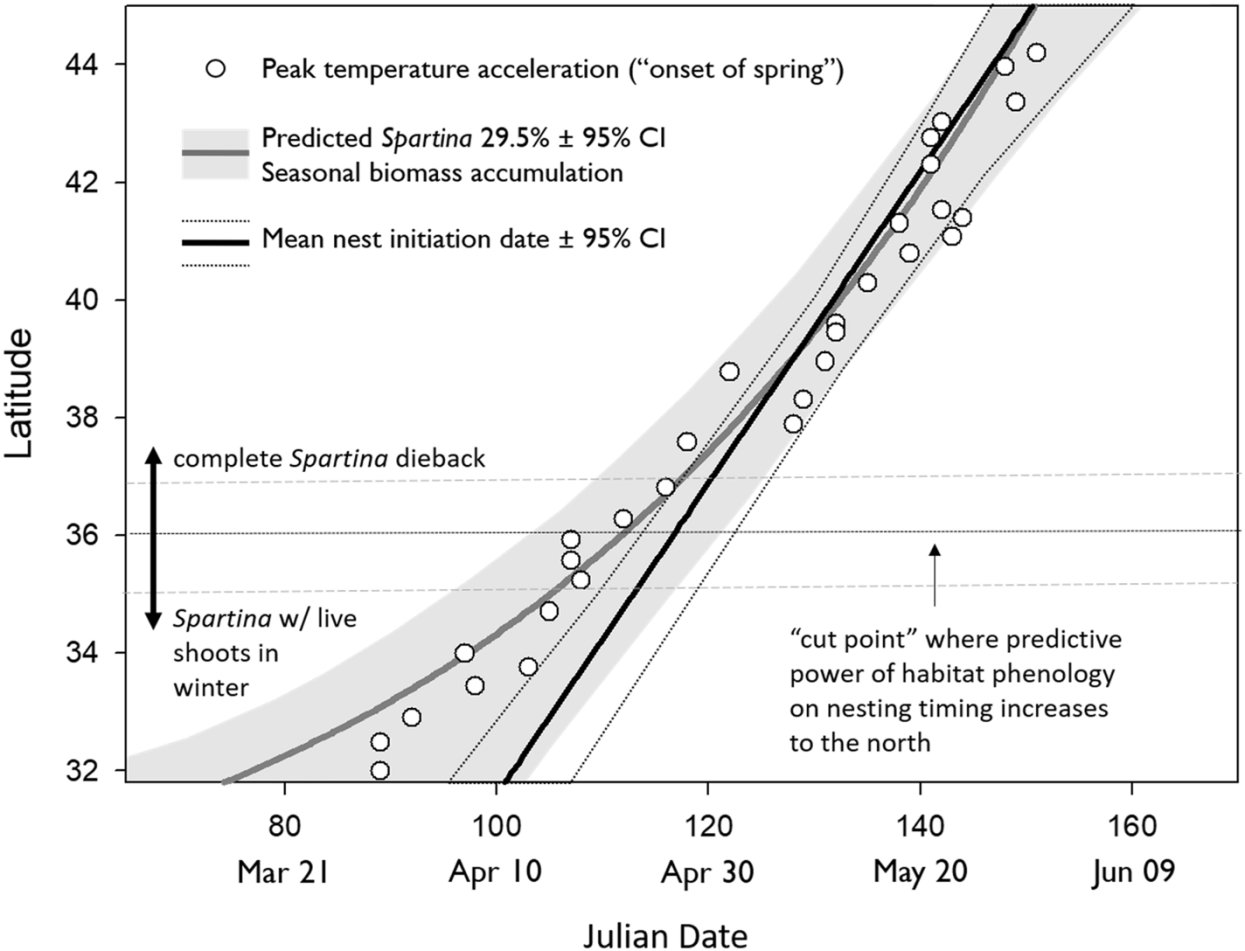
The correlation between two phenological indicators of willet nest initiation: the date of peak spring temperature acceleration (GDD jerk) and the date when Spartina growth reaches 29.5% of seasonal cumulative biomass (see Fig. 3). The regression line from Fig. 2 is displayed to show the close correlation between the phenological indicators and nest initiation timing above 36° latitude. The correlation between the indicators and nesting timing diverges south of this threshold. This threshold corresponds with a phenological shift in plant growth where a warmer climate at southern latitudes allows *Spartina* to maintain green shoots throughout the winter.

There was a significant correlation between the two phenological indicators (temperature and *Spartina* biomass) across the 32–44° latitudinal gradient (slope = 0.95, R^2^ = 0.98, df = 1, *p* < 0.0001, n = 30, Fig. 4, Table S7). Although the phenological metrics were strongly correlated with each other across the entire latitudinal range we examined, they predict nest initiation timing best at the northern latitudes compared to southern latitudes. A cutpoint analysis^75^ of the deviation of peak temperature acceleration date from the predicted date of nest initiation indicates that south of 36° latitude (T_max_ = 4.0429, *p* = 0.0066), the date of maximum spring temperature acceleration (30-year mean) is on average 12 days (± 1.02 SE) earlier than predicted nest initiation date. North of this latitude, this date was a mean ± 3.9 days (± 0.44 days SE) from that of the predicted nest initiation date (Fig. 4) which is similar to the deviation we measured at individual study sites (Fig. 4).

## Discussion

In this study we demonstrate that range-wide timing of migration and breeding events during the annual cycle in a transcontinental migratory bird is shifted progressively later along an increasing latitudinal gradient of breeding sites. This variation in timing is explained by spring salt marsh phenology along the coast of eastern North America. Both temperature and plant-growth derived metrics of spring phenology predicted the timing of mean willet nest initiation nearly to the day across the northern 6° of the 11° latitude gradient we examined.

This gradient in breeding timing had a domino effect, with the timing of all subsequent life stages correspondingly time-lagged with increasing breeding site latitude. This includes post-breeding southbound migration, wintering site arrival, northbound migration and arrival at breeding sites. The singular role of breeding habitat phenology as a driver of life history timing is particularly clear in this study because (1) breeding habitats are composed of the same plant species across a broad latitudinal gradient and (2) individuals across this gradient are using common nonbreeding sites and migratory pathways.

Eastern willets from disparate breeding sites converged on several discrete regions during stopover and wintering periods. The greatest concentration of birds during southbound stopover was along the coast of Suriname, which is known as a major concentration area for willets and other nonbreeding shorebirds^77^, but the northbound stopover along the Southeast U.S. coast was previously unknown as a major concentration point. The highest concentration of wintering birds was along the coast of Maranhão, Brazil. During aerial surveys of wintering shorebirds along the South American coastline in 1980s, Morrison and Ross^77^ likewise recorded the highest abundance of willets and several other shorebird species in the vast estuaries of this region. There was no attempt to distinguish subspecies at the time but our work, along with recent ground-based surveys^78^, confirm that the Atlantic coast of northern South America is primarily occupied by eastern willet rather than western willet (*Tringa semipalmata inornatus*).

Despite using common nonbreeding regions, individuals had varying departure and arrival schedules that corresponded with breeding latitude. It is axiomatic that phenological events occur later further north in temperate latitudes during spring. More notable is our result that this timing trend carries over to subsequent events in the annual cycle, so that more northern birds have a later schedule at all subsequent life stages. Typically, patterns of duration and timing of events in the annual cycle of migratory species are complex, varying according to conditions and constraints at numerous points in this cycle and furthermore varying across a species’ distribution^79^. Consistent lags in timing throughout the annual cycle that are dependent on breeding latitude implies that the duration of all life stages must be consistent regardless of breeding origins. Although the growing season length at salt marsh study sites along this latitudinal gradient between the southeastern and northeastern United States varies considerably, the length of willet nesting season remains the same, with birds making southbound migrations at the peak of salt marsh productivity in mid-summer. Willet breeding season timing is similar to arctic breeding shorebird species where the rapid pace of the breeding season is frequently ascribed to the brief high latitude growing season^80^.

These patterns suggest that even for temperate breeders, although timing is flexible, life stage duration is less so. Overall, annual cycle phenology appears to be balanced in order to maximize time on stationary nonbreeding sites in South America (where all molts occur) while having a breeding season timing that is precisely synced with local phenology in North America. A necessary implication of this pattern is that the timing of arrival at stopover and wintering sites is not synchronized and varies widely based on breeding origin. This is in contrast with other shorebird species which have migration stopovers defined by seasonally pulsed resources that prompt a large proportion of hemispheric populations to converge at a precise time and place regardless of their wintering and breeding origins^81,82^.

The system described here for eastern willet comprises a highly mixed wintering population that segregates across a latitudinal gradient during the breeding season. The population occupying these wintering sites also uses common stopover regions, but the use of these sites is temporally segregated depending on the ultimate breeding latitude of individuals. A similar system where annual cycle timing is determined by latitude for populations using common wintering sites has been demonstrated in an extreme long distance migrant (16,000 km), the bar-tailed godwit (*Limosa lapponica*), breeding in Alaska (60–70° latitude) and wintering in New Zealand^16^ and has also been shown in a passerine^24^, the collared flycatcher (*Ficedula albicollis*). These systems of breeding latitude-dependent timing of the annual cycle suggest that resources required at stopover sites are available and abundant across a broad window of time. Likewise, it suggests that there may be minimal penalty for arriving late at nonbreeding sites relative to other individuals in the population. Despite what appears to be a lack of constraints on arrival timing relative to other breeding populations using the same stopover and wintering sites, breeding is tuned to a latitude-specific timing that all individuals in a given region attempt to achieve, likely to improve prospects of territory acquisition^83^ and reproductive success associated with resource availability^84^ and potentially nesting synchrony among a population^85^.

There may nonetheless be tradeoffs associated with breeding at higher vs lower latitudes. For example, if there is resource competition in nonbreeding areas, then more southern breeding birds that arrive earlier may have access to the highest quality sites first. But late arrival on wintering grounds may be offset by benefits associated with breeding at higher latitudes. For example, higher nest predation at more southern breeding sites has been demonstrated in other shorebird species breeding across a latitudinal gradient at high latitudes^86^ and in salt marshes in our study region^87^. This observation is supported by our results which show that the greatest differentiation in timing across study sites was at nest initiation^88^. Timing differences were least-pronounced for the date of southbound migration departure and winter arrival, likely as a result of variation in reproductive success.

If breeding latitude is the primary determinant of annual cycle timing in temperate regions, this suggests that timing may be constrained by spring habitat phenology. The importance of spring green-up to willets may be associated with a need to nest in live vegetation or it is possible that green-up is correlated with food availability^89^, such as *Uca* crabs^34,76^ which also become active at 10 °C^90^. Both indicators of spring phenology (temperature-derived GDD jerk and *Spartina* biomass accumulation) predicted nesting timing for eastern willets in the northern 6° of our 11° latitude study region (Fig. 4). Although *S. alterniflora* dominates salt marsh vegetation across the gradient, its seasonal growth phenology is more protracted at more southern latitudes. From the southern outer banks of North Carolina southward (i.e. 36° latitude)^66^, *Spartina* plants develop new shoots in fall that grow slowly throughout winter and become the first growth in the following spring^61^. From the northern Outer Banks of North Carolina and northward, *Spartina* dies back completely^61,91^ until growing conditions become favorable in spring when temperatures are above 10 °C^31^.

This divergence of nesting timing and *Spartina* phenology indicates that at more southern latitudes and warmer climates, the timing of willet nesting may not be as constrained by breeding habitat conditions. Instead, physiological considerations (e.g. the time needed to complete molt on the wintering grounds) may take priority as a constraint on willet life history timing in the south, while phenological matching becomes the primary constraint of breeding timing at more northern latitudes. With this the case, we would predict little variation in timing with respect to latitude in southern breeding areas (south of 36°) where annual cycle timing is not delayed by spring green up.

Our findings demonstrate that the timing of annual cycle events in a long-distance migratory bird can be determined by climatic constraints at a single stage within the annual cycle. Likewise, changes in this timing due to climate change are predicted to be governed by phenological trends at breeding sites^92,93^. In species that display more complex spatial patterns of variation in life stage timing and duration, the determinants of phenology may be more numerous and interact with each other. Nonetheless, understanding the interplay between climate and life history phenology is essential for understanding how migratory species respond to environmental conditions that are dynamic in both time and space^94,95^. In particular, identifying the primary environmental drivers of phenology and the life stages during which they occur can help predict the influence of dynamic selection pressures at different phases of the annual cycle which conflict with or complement species’ ability to adapt to environmental change.

## Supplementary information

Supplementary information.
