# Supplementary material for: A green wave of saltmarsh productivity predicts the timing of the annual cycle in a long-distance migratory shorebird
Source: Sci Rep. 2020 Nov 26;10:20658. doi: 10.1038/s41598-020-77784-7 (PMC7693269; doi:10.1038/s41598-020-77784-7)

**Title:** A green wave of saltmarsh productivity predicts the timing of the annual cycle in a long-distance migratory shorebird

**Authors:** Joseph A.M. Smith, Kevin Regan, Nathan W. Cooper, Luanne Johnson, Elizabeth Olsen, Ashley Green, Jeff Tash, David C. Evers, Peter P. Marra


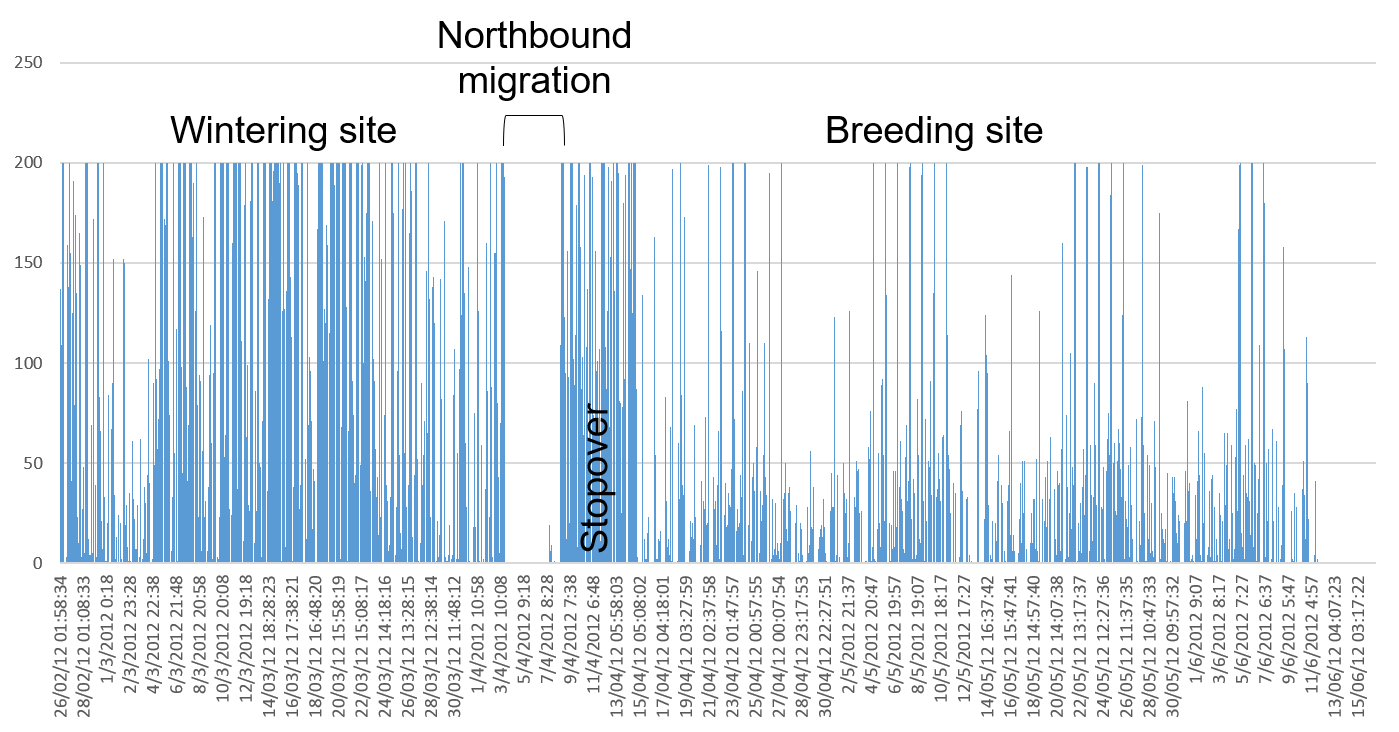


**Figure S1.** Example conductivity data which recorded when the leg-mounted light sensor was immersed in salt water. This image illustrates an extended dry period during northbound transoceanic migration that is uncharacteristic of typical wet-dry signatures observed during sedentary periods.


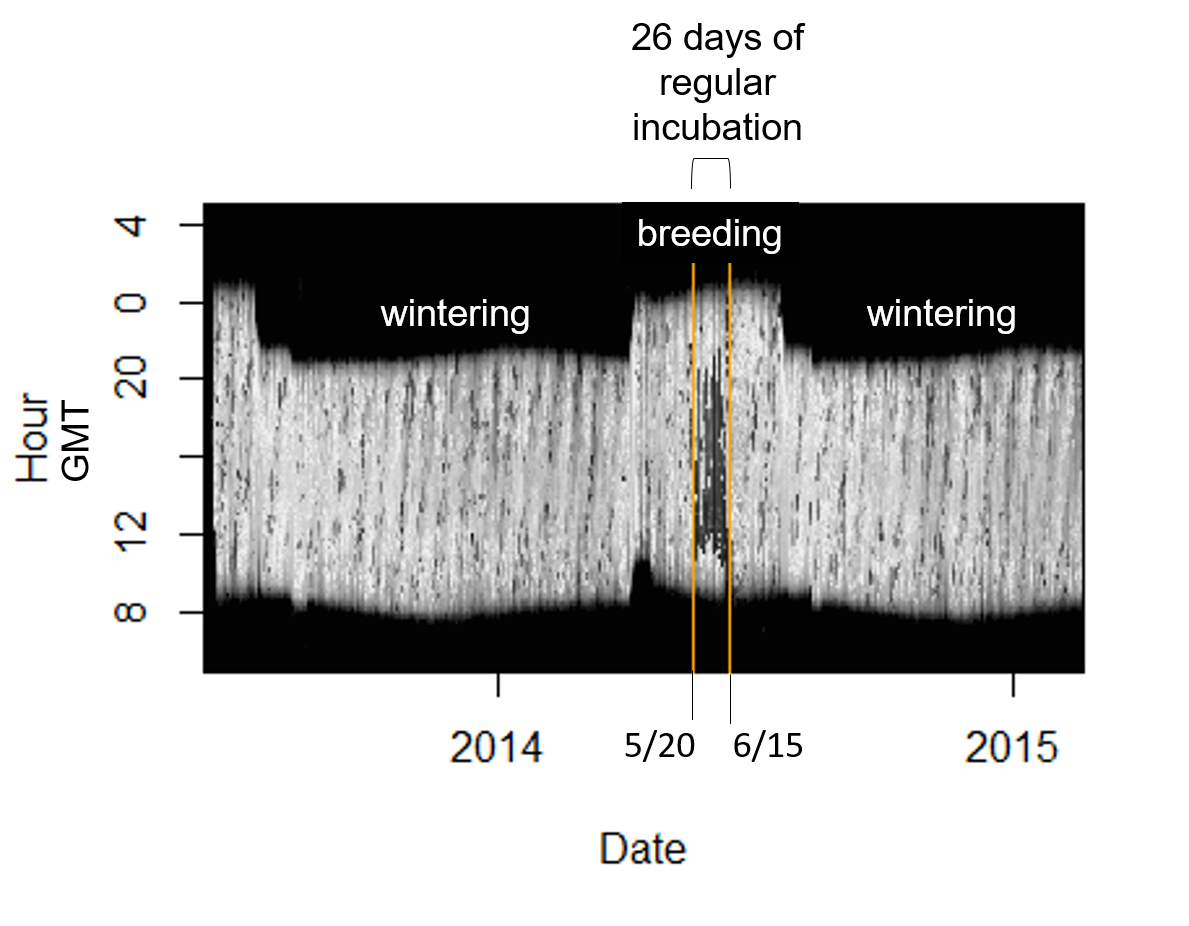


**Figure S2.** Example of geolocator light data where the leg-mounted sensor is obscured during incubation of eggs. Here a period of darkness is depicted each day over a 26-day incubation period.

**Table S1.** Regression parameter estimates for the relationship between latitude and dates of breeding site arrival and incubation initiation derived from geolocators.


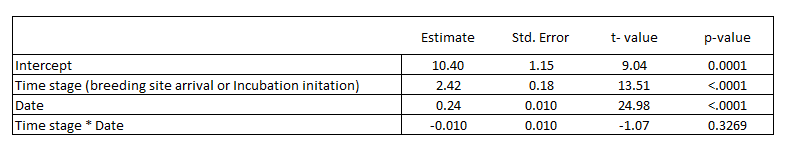


**Table S2.** Regression parameter estimates for the relationship between latitude and breeding site arrival estimated from eBird data.


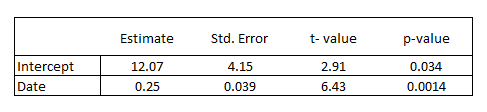


**Table S3.** Regression parameter estimates for the relationship between mean nest initiation date and the date of peak spring temperature acceleration derived from Growing Degree Day data for 5 sites in Maine, Massachusetts, New Jersey, Virginia and Georgia.


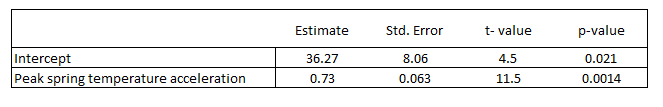


**Table S4.** Regression parameter estimates for the relationship between mean nest initiation date and the date of peak spring temperature acceleration derived from Growing Degree Day data for 4 sites in Maine, Massachusetts, New Jersey, Virginia (Georgia excluded)


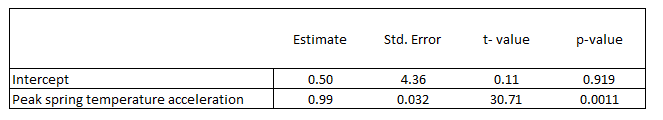


**Table S5.** Regression parameter estimates for the relationship between mean nest initiation date and the date when *Spartina* biomass accumulation reaches 29.5% of seasonal total for 5 sites in Maine, Massachusetts, New Jersey, Virginia and Georgia.


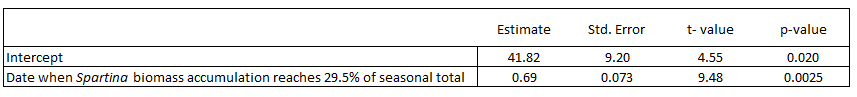


**Table S6.** Regression parameter estimates for the relationship between mean nest initiation date and the date when *Spartina* biomass accumulation reaches 29.5% of seasonal total for 4 sites in Maine, Massachusetts, New Jersey, Virginia (Georgia excluded).


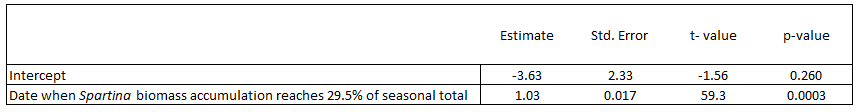


**Table S7.** Regression parameter estimates for the relationship between the date when *Spartina* biomass accumulation reaches 29.5% of seasonal total and the date of peak spring temperature acceleration derived from Growing Degree Day data for 30 sites between Maine and Georgia.


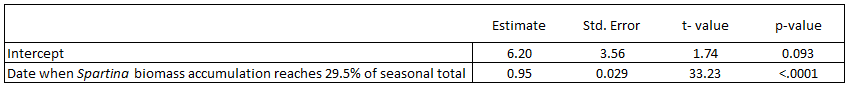

Supplement: Supplementary file 1 — Supplementary information. [file 41598_2020_77784_MOESM1_ESM.docx]
